# Increasing Incidence of *Plasmodium knowlesi* Malaria following Control of *P. falciparum* and *P. vivax* Malaria in Sabah, Malaysia

**DOI:** 10.1371/journal.pntd.0002026

**Published:** 2013-01-24

**Authors:** Timothy William, Hasan A. Rahman, Jenarun Jelip, Mohammad Y. Ibrahim, Jayaram Menon, Matthew J. Grigg, Tsin W. Yeo, Nicholas M. Anstey, Bridget E. Barber

**Affiliations:** 1 Infectious Diseases Unit, Department of Medicine, Queen Elizabeth Hospital, Kota Kinabalu, Sabah, Malaysia; 2 Department of Medicine, Queen Elizabeth Hospital, Kota Kinabalu, Sabah, Malaysia; 3 Ministry of Health, Putrajaya, Malaysia; 4 Sabah Department of Health, Kota Kinabalu, Sabah, Malaysia; 5 Global Health Division, Menzies School of Health Research and Charles Darwin University, Darwin, Northern Territory, Australia; 6 Royal Darwin Hospital, Darwin, Northern Territory, Australia; Eijkman-Oxford Clinical Research Unit, Indonesia

## Abstract

**Background:**

The simian parasite *Plasmodium knowlesi* is a common cause of human malaria in Malaysian Borneo and threatens the prospect of malaria elimination. However, little is known about the emergence of *P. knowlesi*, particularly in Sabah. We reviewed Sabah Department of Health records to investigate the trend of each malaria species over time.

**Methods:**

Reporting of microscopy-diagnosed malaria cases in Sabah is mandatory. We reviewed all available Department of Health malaria notification records from 1992–2011. Notifications of *P. malariae* and *P. knowlesi* were considered as a single group due to microscopic near-identity.

**Results:**

From 1992–2011 total malaria notifications decreased dramatically, with *P. falciparum* peaking at 33,153 in 1994 and decreasing 55-fold to 605 in 2011, and *P. vivax* peaking at 15,857 in 1995 and decreasing 25-fold to 628 in 2011. Notifications of *P. malariae/P. knowlesi* also demonstrated a peak in the mid-1990s (614 in 1994) before decreasing to ≈100/year in the late 1990s/early 2000s. However, *P. malariae/P. knowlesi* notifications increased >10-fold between 2004 (n = 59) and 2011 (n = 703). In 1992 *P. falciparum*, *P. vivax* and *P. malariae*/*P. knowlesi* monoinfections accounted for 70%, 24% and 1% respectively of malaria notifications, compared to 30%, 31% and 35% in 2011. The increase in *P. malariae*/*P. knowlesi* notifications occurred state-wide, appearing to have begun in the southwest and progressed north-easterly.

**Conclusions:**

A significant recent increase has occurred in *P. knowlesi* notifications following reduced transmission of the human *Plasmodium* species, and this trend threatens malaria elimination. Determination of transmission dynamics and risk factors for knowlesi malaria is required to guide measures to control this rising incidence.

## Author Summary

The simian parasite *Plasmodium knowlesi* is a common cause of malaria in Malaysian Borneo; however, little is known about its emergence over time, particularly in Sabah. We reviewed all available Sabah Department of health malaria notification records from 1992–2011, and considered notifications of *P. malariae* and *P. knowlesi* as a single group due to their microscopic similarity. We found that malaria notifications in Sabah have decreased dramatically, with *P. falciparum* and *P. vivax* notifications peaking at 33,153 and 15,877 respectively during 1994–1995, and falling to 605 and 628 respectively in 2011. Notifications of *P. malariae/P. knowlesi* fell from a peak of 614 in 1994 to ≈100/year in the late 1990s/early 2000s, however increased >10-fold between 2004 (*n* = 59) and 2011 (*n* = 703). In 1992 *P. falciparum*, *P. vivax* and *P. malariae*/*P. knowlesi* monoinfections accounted for 70%, 24% and 1% respectively of malaria notifications, compared to 30%, 31% and 35% in 2011. The increase in *P. malariae*/*P. knowlesi* notifications occurred state-wide, appearing to have begun in the southwest and progressed north-easterly. This significant recent increase in *P. knowlesi* notifications following reduced transmission of the human *Plasmodium* species threatens malaria elimination; further research is required to determine transmission dynamics and risk factors for knowlesi malaria.

## Introduction

Malaria elimination is now a goal of many countries in Southeast Asia and the Western Pacific, and large reductions in malaria prevalence have been achieved [1]. However, significant challenges remain, and while the threat of artemisinin resistance has been the focus of much international concern, zoonotic malaria species have received less consideration. Malaysia has had one of the most successful malaria control programs in the region, and aims to be malaria-free by 2020 [1], [2]. However, the simian parasite *Plasmodium knowlesi*, transmitted by the forest-dwelling *Anopheles leucosphyrus* group of mosquitoes, is now a common cause of human malaria in the eastern states of Sabah and Sarawak, and presents an increasing threat to malaria elimination [3], [4], [5], [6], [7].

Documentation of the emergence of this species over time is limited by the inability to distinguish *P. knowlesi* from *P. malariae* by microscopy. Although the first naturally acquired case of human knowlesi malaria was reported from Peninsular Malaysia in 1965 [8], with a second probable case several years later [9], it was not until the early 2000s that a large focus of human infections was described in Kapit, Sarawak [10]. Since this time an increasing number of cases have been reported, and *P. knowlesi* is now the most common cause of human malaria in several districts throughout Sabah and Sarawak [3], [4], [5], [6]. The highest proportion has been reported at Kudat District Hospital (KDH), on the northeast tip of Sabah, where 87% of patients admitted with malaria in 2009 were infected with *P. knowlesi* [3].

Whether this apparent increase in cases however is due to a true emergence of the species or increasing recognition remains uncertain. Evolutionary analyses of sequence data from samples obtained from Sarawak indicate that *P. knowlesi* existed in macaques in Southeast Asia more than 100,000 years ago, with infection in humans likely occurring from the time of human arrival in the region [11]. In the earliest documented malaria survey conducted in Sarawak, in 1952, one third of all malaria cases were reported as *P. malariae* by microscopy [12]. Given the evidence of very few cases of *P. malariae* in Sarawak when PCR methods are used [4], [5], [10], it seems likely that at least some of these cases were *P. knowlesi*. When PCR was performed on the earliest *P. malariae* slides available, taken in 1996, 35/36 (97%) were positive for *P. knowlesi*, with only one being positive for *P. malariae* [13]. In 1999, “*P. malariae*” accounted for 9% of all malaria notifications in Sarawak, and 20% of cases in the Kapit district [14].

In Sabah, limited available evidence suggests that the situation may differ from that of Sarawak, and that *P. knowlesi* infection in humans may have increased only recently. In 2001, only 96/6050 (1.6%) malaria slides referred to the Sabah State Public Health Laboratory were diagnosed as *P. malariae* monoinfection by microscopy, with the proportion increasing to 59/2741 (2.2%) in 2004 [15]. In contrast, microscopy-diagnosed “*P. malariae*” accounted for 621/1872 (33%) of malaria cases reported to the Sabah Department of Health in 2011 (unpublished data from Sabah Department of Health records).

In this study, we reviewed the Sabah Department of Health records of malaria notifications from 1992–2011, in order to investigate the trend of each malaria species over time, and in particular to determine if *P. knowlesi* represents an emerging infection in humans.

## Methods

### Ethics statement

The study was approved by the Medical Research Sub-Committee of the Malaysian Ministry of Health and the Menzies School of Health Research, Australia. All data analysed were anonymised.

### Study site

The north-eastern Malaysian state of Sabah has an area of 73,600 km^2^ and a population of 3.2 million [16]. Situated between 4° and 7° north of the equator, Sabah has a mostly tropical climate, with high humidity and rainfall throughout the year and temperatures of 25–35°C. The southwest interior of Sabah is mountainous, with the Crocker Range separating west coast lowlands from the rest of the state and extending north to Mount Kinabalu at 4095 meters above sea level. Sabah was previously covered almost entirely in dense primary rainforest, however extensive deforestation occurred throughout the 1970s and 1980s, reducing forest cover to 44–63% of the state [17], [18], [19]. Cleared areas have been partly replaced by plantations, with palm oil estates comprising 16% of Sabah's land area [19].

Malaysia has a long history of malaria control programs dating back to the early 1900s, with an initial focus on environmental management techniques. The launch of the Malaria Eradication Program in 1967, followed by state-wide malaria control programs during the 1970s and 1980s, led to large reductions in malaria prevalence, with cases falling from 240,000 in 1961 to around 50,000/year during the 1980s [20], [21]. Further scale-up of malaria control activities began in 1992, consisting of increased surveillance, vector control, training of community volunteers, and early diagnosis and treatment [21]. Use of insecticide-treated nets and indoor residual spraying was implemented in 1995, with nation-wide coverage of the high-risk population reported to be >50% and 25–50% respectively in 2010 [1]. In addition, Malaysia reports 100% confirmatory testing of suspected malaria cases and mandatory notification of detected cases [1].

Mosquito vectors in Sabah include *An. balabacensis* and *An. donaldi* [22], and the *P. knowlesi* hosts, the long-tailed and pig-tailed macaques, are found throughout the state.

### Review of malaria notification records

In Sabah mandatory reporting of all malaria cases to the Sabah State Health Department is generally done by nursing staff, with species normally reported according to microscopy results. Blood slides with parasites resembling *P. malariae/P. knowlesi* are mostly reported, and hence notified, as *P. malariae*.

We reviewed all available malaria notification records held by the Sabah State Health Department. Hard copy summaries of annual malaria notifications by species and by district were available from 1992. From 2007 yearly Excel databases were also available that included limited demographic/epidemiological information for each malaria notification. We therefore recorded the number of notifications of each *Plasmodium* species annually for each district in Sabah from 1992–2011, in addition to the age and sex distribution and seasonal variation of each species from 2007–2011.

### Data analysis

Data were analysed using Stata statistical software, version 10.0 (StataCorp LP, College Station, TX, USA). Spearman's correlation coefficient was used to analyse the association between annual notification rates of the *Plasmodium* species. Median ages were compared using Wilcoxon rank-sum test, and proportions were assessed using the Chi-square test. Edwards' test was used to assess seasonality of the *Plasmodium* species.

Notifications of *P. malariae* and *P. knowlesi* were considered as a single group (“*P. malariae*/*P. knowlesi*”), due to the inability to distinguish these species by microscopy. Mixed-species infections were recorded as a single group, with analysis of these cases limited to annual notification rates.

## Results

### Malaria trends in Sabah state, 1992–2011

**Figure 1:**
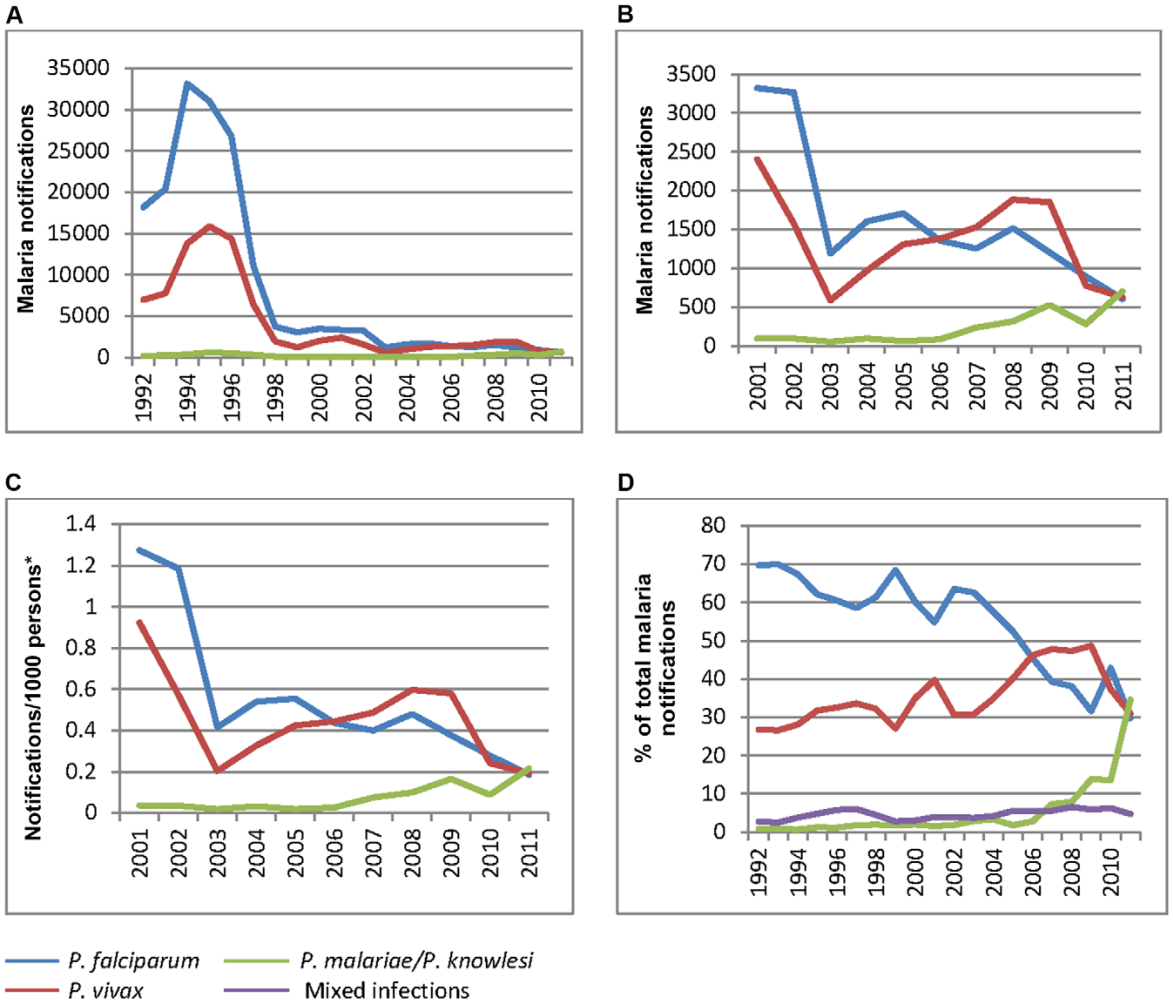
Malaria trends in Sabah, 1992–2011. **A.** Annual malaria notifications by species 1992–2011; **B.** Annual malaria notifications by species 2001–2011; **C.** Malaria incidence by species 2001–2011; **D.** Annual notifications of species as a percentage of total malaria notifications, 1992–2011. *Population projections based on adjusted 2000 and 2010 population [24].

**Figure 2:**
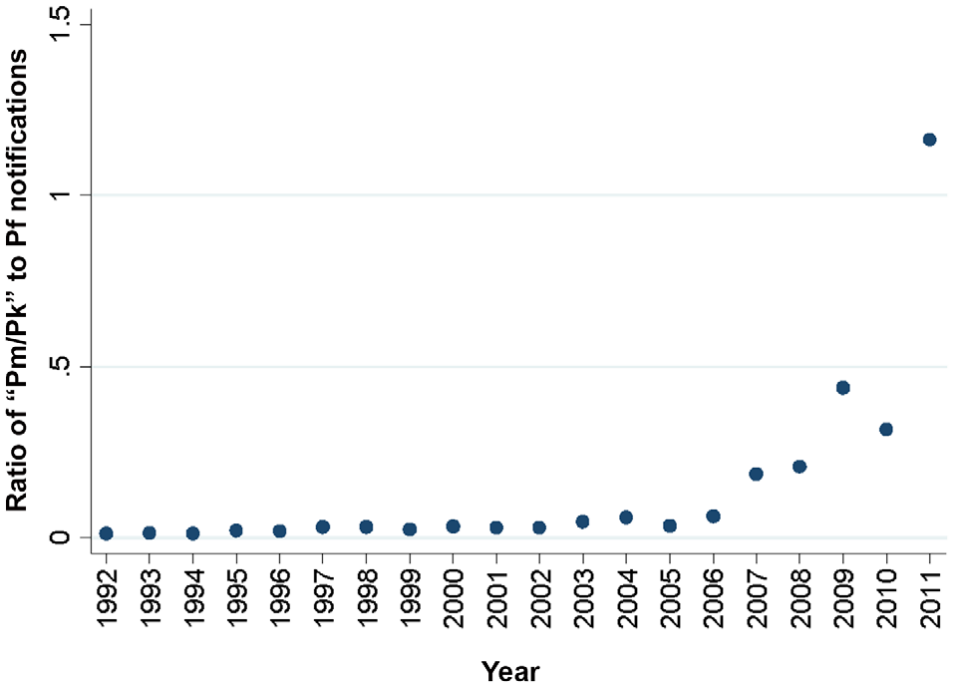
Ratio of *P. malariae/P. knowlesi* to *P. falciparum* notifications, 1992–2011. Pm/Pk = *P. malariae/P. knowlesi*, Pf = *P. falciparum*.

Between 1992 and 2011 the total number of malaria notifications to the Sabah State Health Department decreased dramatically, with *P. falciparum* notifications peaking at 33,153 in 1994 and decreasing 55-fold to 605 in 2011, while *P. vivax* notifications peaked at 15,857 in 1995 and decreased 25-fold to 628 in 2011 (Figure 1). Notifications of *P. malariae*/*P. knowlesi* also demonstrated a peak in the mid-1990s (increasing from 200 in 1992 to 614 in 1994), before decreasing to around 100/year in the late 1990s and early 2000s. Until 2003, annual notifications of *P. malariae*/*P. knowlesi* strongly correlated with those of *P. falciparum* (Spearman's correlation coefficient 0.94, p<0.0001; Figure 2). However, the relationship between the species began to change in the early 2000s, with *P. falciparum* notifications steadily decreasing (from 3264 in 2002 to 605 in 2011) while *P. malariae*/*P. knowlesi* notifications remained stable from the late 1990s to 2006, and then increased markedly from 2007 (Figure 1.B). An inverse correlation was demonstrated between *P. falciparum* notifications and *P. malariae*/*P. knowlesi* notifications between 2004 and 2011 (Spearman's correlation coefficient −0.76, p = 0.028; Figure 2).

Notifications of *P. vivax* generally correlated with those of *P. falciparum* (Spearman's correlation coefficient from 1992–2011 = 0.90, p<0.0001), and with *P. malariae*/*P. knowlesi* notifications until around 2008 (Spearman's correlation coefficient 0.91, p<0.0001). Since 2008 *P. vivax* notifications decreased while *P. malariae*/*P. knowlesi* notifications increased, although this relationship was not statistically significant.

Using Sabah population estimates based on the 1991, 2000 and 2010 Population and Housing Censuses of Malaysia [23], [24], the incidences of *P. falciparum* and *P. vivax* peaked at 16.0 and 7.36/1000 people/year respectively during 1994–1995, and decreased to 0.18 and 0.19/1000 people respectively in 2011 (Figure 1.C). In contrast the incidence of *P. malariae*/*P. knowlesi* peaked at 0.28/1000 people in 1995, decreased to ≈0.02–0.04/1000 people from 2000–2006, and increased to 0.21/1000 people in 2011.

The relative proportions of the *Plasmodium* species changed significantly over the past two decades, with *P. falciparum*, *P. vivax* and *P. malariae*/*P. knowlesi* monoinfections accounting for 70%, 24% and 1% respectively of total malaria notifications in 1992, compared to 30%, 31% and 35% in 2011 (Figure 1.D). A total of 4.4% of all malaria notifications were mixed-species infections, with this percentage increasing slightly over the years from a median of 3.98% from 1992–2001 to 5.20% from 2002–2011 (p = 0.049).

### Malaria trends by district, 1992–2011

**Figure 3:**
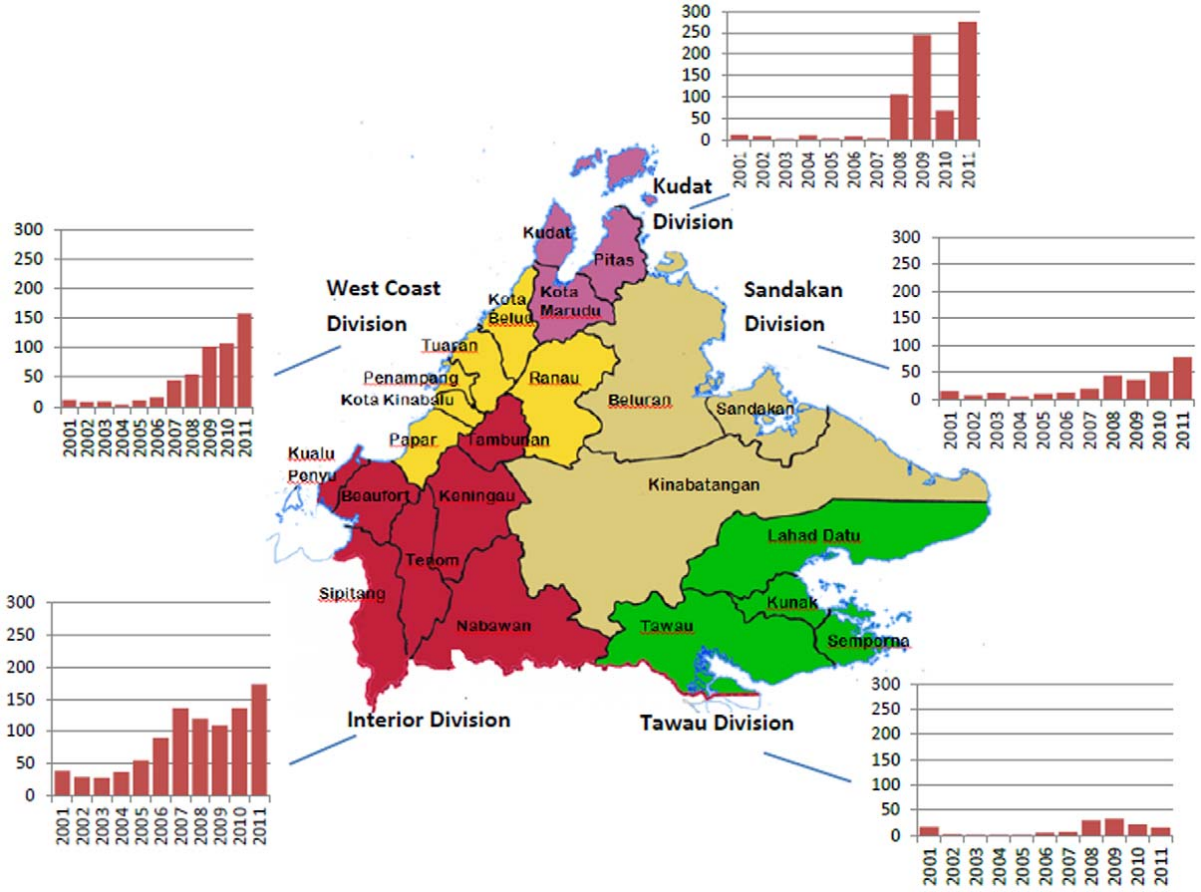
*P. malariae/P. knowlesi* notifications by division. Map shows districts and divisions of Sabah. Bar graphs show annual *P. malariae*/*P. knowlesi* notifications, by division, from 2001–2011. Population of Sabah Divisions in 2010 [37]: Interior 424,524; West Coast 1,011,725; Kudat 192,457; Sandakan 702,207; Tawau 819,955.

**Figure 4:**
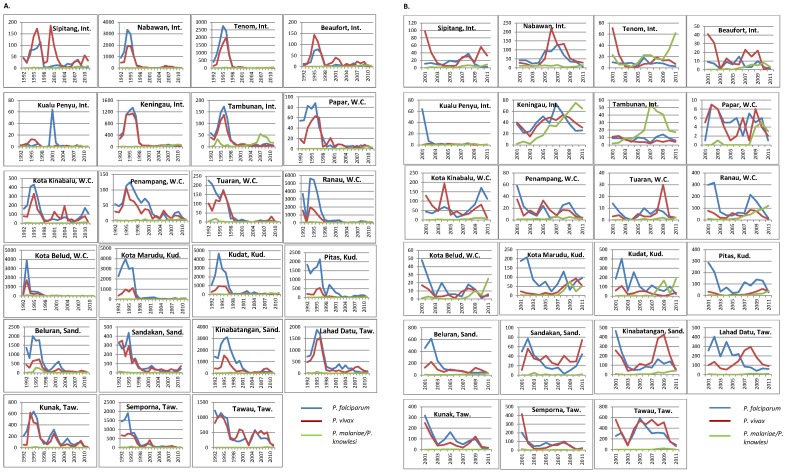
Malaria notifications by district. **A.** Malaria notifications by district 1992–2011; **B.** Malaria notifications by district 2001–2011. Int.: Interior Division; W.C.: West Coast Division; Kud.: Kudat Division; Sand.: Sandakan Division; Taw.: Tawau Division.

The 23 districts of Sabah (Figure 3) in general have experienced similar malaria trends over the past two decades, with *P. falciparum* and *P. vivax* notifications falling dramatically in all districts (Figure 4). *P. malariae*/*P. knowlesi* notifications mostly remained at low stable levels throughout the 1990s, accounting for <5% of total notifications in 87% of district-years from 1992–1999. Exceptions included Tambunan from 1993–1994 and Beluran from 1995–1998, where *P. malariae/P. knowlesi* accounted for 38/255 (15%) and 701/6980 (10%) of malaria notifications respectively, and Tenom from 1998–1999 and Tuaran in 1994 and 1999, where approximately 6% of malaria notifications were *P. malariae/P. knowlesi*.

Since the early 2000s most districts have experienced an increase in notifications of *P. malariae/P. knowlesi* (Figure 3 and Figure 4.B). This increase appears to have begun initially in the Interior Division, in the southwest of the state adjacent to Sarawak, where notifications nearly doubled between 2003 (n = 28) and 2005 (n = 55), and more than doubled between 2005 and 2007 (n = 136), before increasing at a slower rate through to 2011. In the West Coast Division to the northeast notifications appear to have increased later, remaining below 20 per year from 2001–2006 and then increasing to 45 in 2007 and 102 in 2009. Continuing northeast to the tip of Borneo, Kudat Division has experienced the most remarkable and recent increase in *P. malariae/P. knowlesi* notifications, with cases increasing from 2–11 per year from 2001–2007, to 106 in 2008, 245 in 2009, and 276 in 2011. In the eastern districts of Sabah (Sandakan and Tawau Division) notifications of *P. malariae/P. knowlesi* have been fewer, although have been increasing since 2008.

In 2011 Kudat district accounted for the highest number of *P. malariae/P. knowlesi* notifications (184, 26%), followed by Ranau (121, 17%), Keningau (65, 9%), Tenom (62, 9%) and Kota Marudu (52, 7.4%).

### Age and sex distribution of malaria notifications

**Figure 5:**
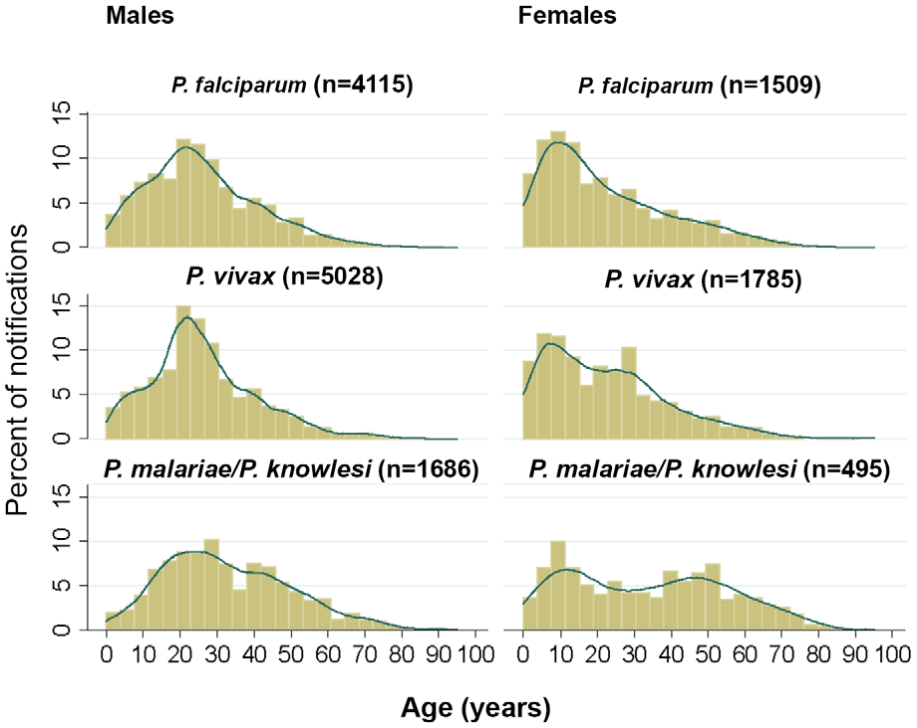
Age distribution of *P. falciparum*, *P. vivax* and *P. malariae*/*P. knowlesi*, 2007–2011.

Epidemiological characteristics of notifications according to species were assessed from 2007–2011, when relevant data were recorded for each notification. This time period included 16,011 malaria notifications, although species was not recorded for 373 (2.3%). The overall median age of patients with *P. malariae/P. knowlesi* (31 years) was significantly higher than that of patients with *P. vivax* or *P. falciparum* (median ages 23 years for both, p = 0.001). Males with *P. malariae*/*P. knowlesi* demonstrated an approximately normal age distribution, with a mean, median and interquartile range of 33, 30 and 20–45 years respectively (Figure 5). In contrast females with *P. malariae*/*P. knowlesi* appeared to demonstrate a bimodal age distribution, with local maxima at 9–12 and 50 years (Figure 5). While most males (71%) with *P. malariae*/*P. knowlesi* were between the ages of 15 and 50 years, with 13% of cases occurring in children <15 years and 17% occurring in adults >50 years, only half (50%) of female cases were aged 15–50 years, with 28% occurring in children <15 years old and 24% occurring in adults >50 years. Among adults (≥15 years) with *P. malariae/P. knowlesi*, females were significantly older than males (median age 43 years vs. 33 years, p<0.0001).

Among patients with *P. vivax* and *P. falciparum* the overall median age was lower among females than it was among males (median age 20 and 24 years for females and males respectively with *P. vivax*, p<0.0001; and 17.5 and 24 years for females and males respectively with *P. falciparum*, p<0.0001). As with *P. malariae*/*P. knowlesi* however, adult females with *P. vivax* were older than adult males (median ages 30 and 27 years respectively, p = 0.002).

The median age of all malaria patients increased progressively from a median of 24 years in 2007 to 27 years in 2011 (Spearman's correlation coefficient 0.04, p<0.0001). The proportion of patients >50 years old also increased, from 244/3191 (7.7%) in 2007, to 364/4135 (8.8%), 345/4009 (8.6%), 244/2644 (9.2%) and 263/2032 (12.9%) in the years 2008, 2009, 2010 and 2011 respectively (p<0.0001). Among patients >50 years old, *P. malariae/P. knowlesi* cases as a proportion of all malaria notifications increased from 43/244 (17.6%) in 2007 to 131/263 (49.8%) in 2011 (p<0.0001).

A greater proportion of patients with *P. malariae/P. knowlesi* were male (77% compared to 73% of patients with *P. vivax* and *P. falciparum*, p = 0.0007), and this proportion increased among those aged 15–60 years, of whom 82% were male, compared to 63% outside this age range (p<0.0001).

### Seasonal variation

**Figure 6:**
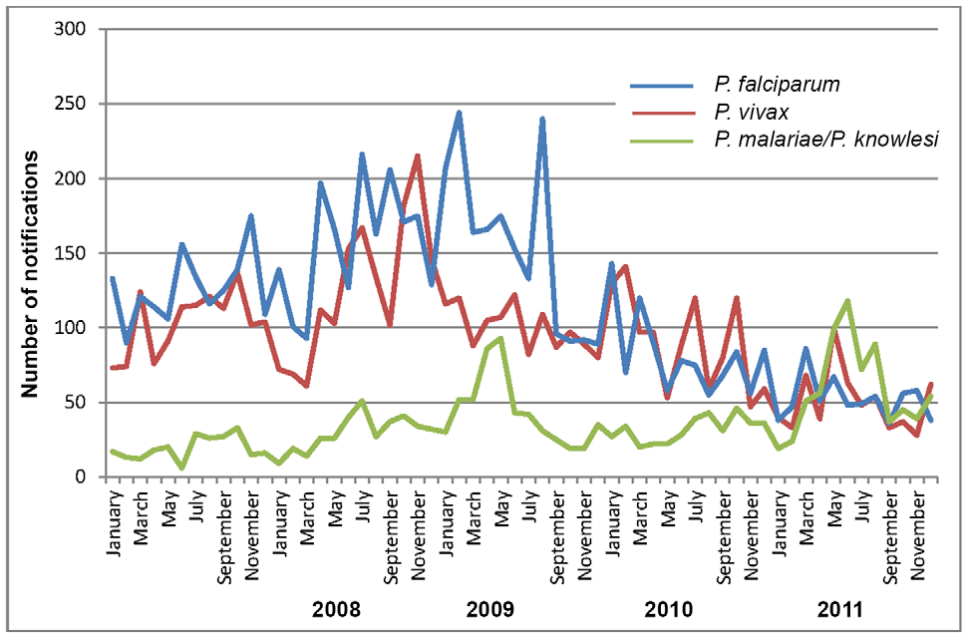
Monthly malaria notifications, 2007–2011.

From 2007–2011 significant seasonality was demonstrated for all *Plasmodium* species, with maximum notifications occurring in July, April and June for *P. falciparum* (p = 0.0001), *P. vivax* (p = 0.002) and *P. malariae/P. knowlesi* (p = 0.0001) respectively (Figure 6).

## Discussion

Although *P. knowlesi* is now well documented in Sabah, the emergence of this species over time has not been previously described. In this study, we found that while cases of *P. knowlesi* (reported as “*P. malariae*”) may have been prevalent at low levels for decades, a significant increase in notifications has occurred over the past decade. This increase follows a dramatic reduction in notification rates of *P. vivax* and *P. falciparum*. In fact over the past decade, a strong inverse correlation has occurred between notification rates of *“P. malariae/P. knowlesi”* and *P. falciparum*.

Available evidence does not allow us to determine what proportion of “*P. malariae/P. knowlesi*” notifications during the last two decades is actually *P. knowlesi*, with PCR testing only instituted at the Sabah State Reference Laboratory in 2005 [15], and no PCR results available from Sabah blood samples prior to 2003 [4]. In the 1990s when prevalence of *P. falciparum* and *P. vivax* was high, it is possible that a significant number of *P. malariae* cases also occurred. However recent studies demonstrate that, at least since 2007, PCR-confirmed *P. malariae* in Sabah is rare. Although eight cases of *P. malariae* were detected by PCR from 49 “*P. malariae*” blood films taken from Sabah during 2003–2005 (with six of these from Kudat) [4], four subsequent studies identified only eight (0.6%) PCR-confirmed *P. malariae* infections among 1286 patients with PCR-confirmed *Plasmodium* infection in Sabah from 2007 to 2011 [6], [7], [15], [25]. In one of these studies only four (0.8%) *P. malariae* infections were identified from 475 patients with PCR-confirmed *Plasmodium* infections in Kudat from 2009–2011, including 365 with microscopy-diagnosed “*P. malariae*” [25]. In another, *P. malariae* was detected by nested PCR in only two of 318 (0.6%) microscopy-diagnosed *P. malariae* cases referred to the Sabah State Public Health Laboratory in 2009 [15]. Furthermore, the age and sex distributions of “*P. malariae/P. knowlesi*” notifications since 2007 in the current study are very similar to those described in a previous study in Kudat, in which 345 patients with PCR-confirmed *P. knowlesi* were analysed [25]. Given the unique age distribution of *P. knowlesi*, this strongly suggests that a large majority of “*P. malariae/P. knowlesi*” notifications, at least since 2007, are indeed *P. knowlesi* cases.

The reason for the older age group affected by *P. knowlesi* in this and previous studies [5], [7], [25] remains unclear, however may relate to greater forest exposure among older individuals, with farmers and plantation workers over-represented in this age group [7]. The bimodal age distribution of females affected by *P. knowlesi* requires further investigation, but may possibly relate to lower forest exposure among young adult females; this may also account for the finding in this and other studies [7], [25] that, among adults with knowlesi malaria, females are older than males. Concurrent zoonotic and human-human transmission may also explain a bimodal age distribution.

There are several possible explanations for the emergence of *P. knowlesi*. Firstly, increased recognition of the species may account for increased reporting by microscopists. Although this possibility cannot be excluded, the previous high prevalence rates of malaria in Sabah ensured that microscopy skill levels were maintained at high levels. It seems unlikely therefore that large numbers of “*P. malariae*” slides would have been misdiagnosed as *P. viva*x or *P. falciparum*. In fact, in a study involving blood films obtained from 243 patients with PCR-confirmed *P. knowlesi* in Sarawak between 2001–2006, only 4.5% and 6.6% were misdiagnosed by microscopy as *P. falciparum* and *P. vivax* respectively [4], and it is likely that a majority of these blood films would have been reported prior to the increased awareness of *P. knowlesi*. The consistency of notification trends across districts further supports the overall reliability of the microscopy reports and the State Department records. Furthermore, the number and proportion of all malaria patients aged >50 years increased significantly between 2007 and 2011. Given that this age group is over-represented among patients with knowlesi malaria [7], [25], this finding is consistent with a true increase in the proportion of *P. knowlesi* cases and cannot be attributed to increased recognition.

We believe, therefore, that the prevalence of *P. knowlesi* in Sabah has increased, and that this has occurred as a result of environmental change together with reducing rates of the other human malaria species. The extensive deforestation that has occurred in Sabah has led to encroachment of humans into previously forested areas, resulting in increased interaction with mosquito vectors and simian hosts. Furthermore, the removal of habitat together with malaria control activities may have led to a change in vector behaviour, or a vector shift, as has been seen in the Kinabatangan region where the previously dominant malaria vector *An. balabacensis* appears to have been displaced by *An. donaldi* [22]. Both these factors may increase the chance of human acquisition of *P. knowlesi*, although further research regarding *P. knowlesi* vectors in Sabah is needed.

Finally, the finding in this study that the prevalence of *P.knowlesi* appears to have increased very recently, long after Sabah's most extensive period of deforestation during the 1970s and early 1980s [17], suggests that decreasing rates of *P. vivax* and *P. falciparum* are likely to have contributed directly to this trend. Possible explanations for this may be derived from examining the relationship between *P. falciparum* and *P. vivax*, as in other regions prevalence of *P. vivax* has increased as rates of *P. falciparum* decrease [26], [27]. In addition, studies of *P. vivax* and *P. falciparum* have demonstrated lower than expected rates of mixed infections [28] and the occurrence of reciprocal seasonality between the two species [29]. These observations suggest an inhibitory interaction between *P. falciparum* and *P. vivax*, a phenomenon also demonstrated in early syphilis studies in which *P. falciparum* was found to suppress *P. vivax* parasitemia when both species were inoculated simultaneously [30], [31]. More recently, Bruce et al. reported that asymptomatic children living in a highly endemic area demonstrated relatively stable total parasite density counts despite changes in the density of individual species, suggesting density-dependent regulation that transcends species [28]. Similar interactions between *P. knowlesi* and either *P. falciparum* or *P. vivax* may explain the malaria trends in Sabah, with density-dependent regulation possibly accounting for previously low rates of symptomatic *P. knowlesi*. The occurrence of density-dependent regulation may also explain the lack of earlier reports of severe “*P. malariae*”, similar to reports from other regions that cases of severe vivax malaria increased as the prevalence of *P. falciparum* reduced [27].

In addition, it is possible that cross-species immunity may play a role in the malaria prevalence patterns observed in Sabah. Although heterologous immunity does not generally occur between human malaria species, it has been argued that a degree of cross-resistance may be more likely to occur between species infecting different hosts [32]. In a study involving sera from Gambian adults highly immune to *P. falciparum*, antibodies were found to bind to the surface of *P.knowlesi* merozoites, although erythrocyte invasion was not prevented [33]. In addition, data from neurosyphilis malariotherapy series demonstrated that patients who had been previously infected with *P. vivax* were less susceptible to infection with *P. knowlesi* [34]. Loss of cross-protection provided by immunity to *P. falciparum* or *P. vivax* may be particularly relevant given that *P. knowlesi* tends to effect older individuals; frequent exposure to *P. falciparum* and *P. vivax* may previously have protected this age group from infection with *P. knowlesi*.

The finding that notification rates of *P. knowlesi* have increased following decreasing prevalence of the other malaria species has implications for malaria control in any country where *P. knowlesi* is known to occur, which includes nearly every country in Southeast Asia [35]. In Sabah, *P. knowlesi* is now the most common cause of malaria, and based on current trends, is likely to become increasingly dominant and may extend to previously unaffected districts. Furthermore, human-to-human transmission, if not already occurring, may become more likely as prevalence continues to increase. Close monitoring of *P. knowlesi* in Sabah and elsewhere is therefore essential, including accurate reporting of microscopy-diagnosed “*P. malariae*” as *P. knowlesi*, as has been previously recommended [4], [36], in addition to PCR-confirmation of suspected cases. Moreover, further research is required to determine the risk factors for knowlesi malaria, in order that malaria control programs can include strategies to address the increasing prevalence of this species. Although Malaysia has been highly successful in reducing rates of *P. falciparum* and *P. vivax*, malaria elimination will not be achieved unless control of knowlesi malaria is addressed.
